# The Intermittent Infectiousness of Scarlet Fever

**Published:** 1909-03-06

**Authors:** William Butler

**Affiliations:** Medical Officer of Health for Willesden


### THE INTERMITTENT INFECTIOUSNESS OF SCARLET FEVER.

By WILLIAM BUTLER, M.B., C.M. (Glasgow), D.P.H.; Medical Officer of Health for Willesden.

(A Paper read before the Epidemiological Section of the Royal Society of Medicine.)

(*Concluded from page* 558.)

### Return Cases.

If it be possible for the infection, diffused by the patient prior to his removal to hospital, to recrudesce at intervals during at least six weeks after his removal and give rise in those who, apparently in their own persons, have harboured the infection, to attacks of scarlet fever, it is not remarkable that the patients who have actually suffered from the disease should, after their recovery and release from isolation, be found frequently to continue its spread.

Probably no one will now dispute this explanation of the occurrence of return cases. What has not so generally been recognised, however, is that the infecting cases, when discharged from hospital, may apparently be non-infectious, while at a later date they will present unmistakable evidence of an aroused infectious activity. Perhaps the most striking evidence of the truth of this view is to be found in the intervals which elapse between the return home of the infecting case and the occurrence of derivative return cases. I have analysed over ninety cases, accounted for as return cases, occurring in Willesden, in relation to the interval elapsing between the return home of the infecting case and the occurrence of the return case, with the following tabulated results:—

Analysis of 93 Return Cases: showing Interval between Release from Isolation of Infecting Case and onset of Return Case, Number of Cases, and Percentage.

| Interval | No. of Cases | Percentage of Total |
|---|---|---|
| 7 days | 32 | 34.4 |
| 14 ,, | 21 | 22.5 |
| 21 ,, | 9 | 9.6 |
| 28 ,, | 20 | 21.5 |
| Over 1 month | 11 | 11.8 |

It is to be observed that only one-third of the cases are infected within the recognised maximum incubative period, dating from the home-coming of the infecting case, while in nearly 12 per cent. of the cases the onset of illness in the derivative cases is deferred for over a month. It is found upon inquiry that practically in all cases there has been intimate contact between the infecting and return case immediately upon release from isolation of the former and throughout the whole subsequent period. Either the infectivity of the patients released from isolation varies in the interval between their return home and the occurrence of derivative cases, or the resistance of the derivative cases changes critically during the period. Many of the infecting cases at the time of occurrence of the derivative cases are found to be subject to mucous or purulent discharges; and when, as is so frequently the case, it is found that the discharge is of recent origin, it is difficult to escape the conclusion that with the advent of the catarrh there has been a recrudescence of infectivity.

An analysis of the ninety-three return cases already referred to in relation to the presence of discharges yields the following results:—

Analysis of 93 Return Cases: showing Number of Infecting Cases giving a History of "Cold" or Discharge.

| | |
|---|---|
| Number of infecting cases with discharge... | 35.0 |
| Percentage of total ... ... ... ... | 37.6 |

Of the above, in twenty-six of the cases found with discharge or cold upon their return home, all left the hospital free from discharge, but fifteen had had during their stay in hospital either otorrhœa or rhinorrhœa, while ten were free from discharges during their stay in hospital, but were found to be suffering at the time of inquiry from discharge either from nose or ear. One case was a home-isolated case, and was found at the time of inquiry to be suffering from discharge. Thus 60 per cent. of those found with discharges at the time of the occurrence of the return case had already suffered in hospital from discharge, although apparently cured at the time of release from isolation. That 40 per cent. of those suffering from discharge should have been free during their stay in hospital is very remarkable. It is probable that the return to the comparatively unhygienic conditions of home, after the hospital *régime*, renders the patient liable to infections of the mucous membrane, and any previous discharge is more likely to be set up afresh under these conditions.

Obviously, however, the presence of discharges is not accountable in itself for the recurrence of return cases. Many of the infecting cases at the time of communicating the disease present a perfectly normal appearance, and are quite free from pathological discharges. But so, in fact, are many of the cases which during the clinical course of their attack develop one or other of the complications of the disease. Apart from the desquamation many patients convalescent from scarlet fever are perfectly normal to appearance and to physical examination, when an adenitis, a nephritis, or an otorrhœa reveals a lurking infectivity, of the existence of which prior to the onset of the complications there was no evidence. These exacerbations of infective processes in scarlet-fever patients are characteristic, and the clinical course of the disease has its parallel in the epidemiological history in which most typically scarlet fever is revealed. The patient, after perhaps months of segregation, recovers an appearance of normality and is assumed free from infection, but on commingling with those susceptible to attack he communicates the infection either at once or at an indeterminate period subsequent to his apparent recovery. We have seen how the infection, diffused prior to the isolation of the patient, may reverberate as it were in infected households for weeks at least after the removal of the discernible cases. For how long may the infection be diffused after the return to the home of the patients who were the starting-points of such outbreaks? It has been customary to take the somewhat arbitrary period of a month or thereabouts after the date of release from isolation as that during which recurrent cases in the household might be considered "return cases," when no evidence to the contrary is forthcoming. There is probably no more ground for ascribing the limit of infectiousness after release from isolation to this arbitrary period than there was for supposing that the period of six weeks is the natural limitation of infectiousness in ordinary uncomplicated scarlet fever. Yet, as may justly be inferred from consideration of the tables given, both of these periods in relation to their respective cases coincide with the interval during which the greatest volume of infectious spread occurs.

It is now some years ago that I was struck by the frequency with which scarlet fever invaded a house for a second time a year or more after the occurrence of a first case in the household. Such cases have not infrequently been recorded, and explanations have been offered that they were to be accounted for by persistence of the contagium in some article which had escaped disinfection. I have not found that the greatest attention to the details of disinfection by the improved methods of recent years has in any way prevented the recurrence of such cases after prolonged intervals. Accidental coincidence, however, may lend to the occurrence of such cases a striking phenomenalism which may readily be misinterpreted in terms of causal sequence, and which a wider survey of the facts would possibly disprove.

Does scarlet fever recur in houses invaded by the disease with greater frequency than in houses previously free from its occurrence? and, if so, for what period is the increased frequency to be observed, and to what is it to be ascribed? With the view of throwing some light on these questions I have analysed the distribution of scarlet fever in Willesden in relation to previously infected houses over a period of several years—namely, from 1900 to 1907 inclusive. During this time there have been notified 3,857 cases of scarlet fever, occurring in 2,882 houses. Recurrent infections in houses have been distributed according to the intervals shown in the respective columns. These intervals represent the period between the receipt of one notification and that of the next in respect of cases occurring at the same address. All instances in which notifications were received within three weeks of previous notifications of cases from the same house have been ignored. For comparison with the results so obtained, I have calculated the number of cases, in previously infected houses, which during identical comparative periods would have occurred had the incidence upon previously infected houses been the same in the respective periods as upon all the houses in Willesden. That is to say, that the houses yielding the recurrent cases would, in the periods in which they occurred, have furnished the number of cases set out in the comparative figures, had these houses to an equal extent with all the houses in Willesden shared in the distribution of scarlet fever. The figures calculated for comparison are thus corrected for the varying incidence of scarlet fever during the eight years included in order that a completed five years of recurrences might be observed.

It will be noted that the actual incidence upon previously infected houses is higher throughout the whole period than the comparative figure, which represents what may be called the normal indifferent incidence. As the interval between primary and secondary invasion of the house lengthens, however, the difference becomes less. Had it remained constant throughout, the invariable maximum would probably have been wholly accounted for by increased risks which already invaded houses may be supposed to incur owing to age-distribution of their inmates. This must be assumed to be a selective influence in the formation of a class of already infected houses. But the fact that the difference is so much less marked in the groups with recurrences exceeding three years than in the groups with recurrences within this period cannot be explained on this ground. The data are not of sufficient magnitude to warrant anything in the nature of definite conclusions, but they are very suggestive. There is nothing inherently impossible in a recrudescence of infectivity of scarlet fever between two and three years after its onset. It has been observed that return cases are even more frequently associated with patients who have undergone prolonged detention in hospital than with those simpler cases which permit of early discharge.

So soon as it is recognised that return cases do not depend for their infectivity upon hospitalism—a fact which is disproved by their occurrence among home-tiousness, which in these intervals appears to have been quiescent. Such intermittency in the diffusion of the disease does not appear to be confined wholly to those discoverably suffering from it. It would seem necessary, in order to account for many of the facts in the spread of scarlet fever, to postulate "carrier cases" analogous to the demonstrable passive bearers of the diphtheria bacillus and the more recently discovered typhoid carriers. The voiding of typhoid bacilli has been shown to be an intermittent phenomenon in one detected carrier of the diseases,* and an analogous process may be inferred in scarlet fever. There appears to be little ground for seeking an extra-corporeal habitat for the infectious material of scarlet fever if we except the one cultural medium—milk; and a view more consonant with all the facts would seem to be that there is continuously diffused throughout the community the flora—if it be a flora—of scarlatinal infectivity, which under suitable seasonal and other conditions is communicated from person to person in toxic quantity and degree, from time to time appearing phenomenally as sporadic or epidemic scarlet fever.

There appears good ground for saying that the change from a hygienic to a relatively insanitary atmospheric environment, in those who are the hosts of the infection, arouses its kinetic energy; and it is in this way that many hospital return cases are to be accounted for. In the multiple infections of the home, many of them probably non-pathogenic and operative only because of the symbiotic processes they induce, are to be found conditions which offer a promising field of inquiry. The vagaries of scarlet fever will not fit the somewhat stereotyped views with

Table showing Recurrent Cases (in black figures) and Comparative Figures (in plain figures) in Interval Groupings in relation to which the respective houses were Primarily Invaded during an Identical Period.

| Year | 3 Weeks to 3 Months | | 3 to 6 Months | | 6 to 12 Months | | 1 to 2 Years | | 2 to 3 Years | | 3 to 4 Years | | 4 to 5 Years | |
|---|---|---|---|---|---|---|---|---|---|---|---|---|---|---|
| 1900 ... ... | **8** | 0.7 | **6** | 0·9 | **5** | 1.8 | — | — | — | — | — | — | — | — |
| 1901 ... ... | **28** | 1.9 | **5** | 2.5 | **7** | 5.0 | **9** | 6.0 | — | — | — | — | — | — |
| 1902 ... ... | **14** | 1.3 | **5** | 1.7 | **4** | 3.4 | **6** | 8.0 | **6** | 4.9 | — | — | — | — |
| 1903 ... ... | **16** | 1.4 | **9** | 1.8 | **8** | 3.6 | **10** | 6.8 | **4** | 8.0 | **3** | 4.8 | — | — |
| 1904 ... ... | **22** | 0.5 | **4** | 0.7 | **1** | 1.4 | **6** | 4.4 | **5** | 4.1 | **3** | 4.9 | **1** | 3.0 |
| 1905 ... .... | **15** | 0.9 | **8** | 1.1 | **6** | 2.2 | **7** | 3.4 | **11** | 5.5 | **12** | 5.1 | **8** | 6.0 |
| 1906 ... ... | **23** | 2.2 | **11** | 2.7 | **7** | 5.9 | **14** | 7.1 | **14** | 5.5 | **9** | 8.9 | **12** | 8.2 |
| 1907 ... ... | **36** | 2.0 | **6** | 2.5 | **11** | 5.0 | **18** | 10.8 | **8** | 6.5 | **6** | 5.1 | **7** | 8.3 |
| Totals ... | **162** | 10.9 | **54** | 13.9 | **49** | 28.3 | **70** | 46.5 | **48** | 34.5 | **33** | 28.8 | **28** | 25.5 |
| Mean ... ... | **20.25** | 1.4 | **6.75** | 1.7 | **6.1** | 3.5 | **10.0** | 6.6 | **8.0** | 5.7 | **6.6** | 5.72 | **7.0** | 6.4 |

isolated cases—there is no difficulty in conceiving that the case which is admittedly infectious after nine months' isolation in hospital may be found not to have ceased to be infectious twelve months, or even two years, later. Certainly the grosser lesions may persist for longer periods, and such occasional persistence of prolonged infectivity would account for the statistical results with which we are here dealing. It does not follow that such cases are continuously infectious during this time. All the facts seem to indicate that continuous infectiousness is not a feature of scarlet fever. Many cases seem from the first to be little, if at all, infectious, and the temporal distribution of the derivatives from known infecting cases indicates a rapid decline in infectious activity. Yet, after intervals of widely varying range, there is frequently to be observed an irruption of active infec-which it has been extensively identified. They are so elusive of formulation that, of almost every statement which may be made concerning it, the opposite is also true: it is a very slightly infectious disease, it is a highly infectious one; it is a very mild affection, it is a most dangerous disorder; it breeds true, but scarlet fever convalescents frequently communicate diphtheria; its infectiousness is short-lived, but it endures for $x$ months or years. Such paradoxes might be indefinitely multiplied. They merely show the difficulty of formulating a comprehensive statement of the natural features of the disease and of presenting any harmonious concept acceptable as a theoretical account of its varied phenomena.

* See paper on "Typhoid Carriers," by D. S. Davies, M.D., and I. Walker Hall, M.D., Proc. Roy. Soc. Med., 1908, i. Epid. Sec., p. 175.

